# What Distinguishes Males With Sexual Dysfunction Who Present to Either Psychiatrists or Urologists?

**DOI:** 10.7759/cureus.43161

**Published:** 2023-08-08

**Authors:** Metin Yığman, Fatih Yığman

**Affiliations:** 1 Urology, Ankara Etlik Integrated Health Campus, Ankara, TUR; 2 Psychiatry, Independent Scholar, Ankara, TUR

**Keywords:** body image, self esteem, help-seeking behaviours, erectile dysfunction, sexual dysfunction

## Abstract

Background

Organic and psychological causes are intertwined in the etiology of sexual dysfunction (SD). Another important point, as well as the importance of etiology in the treatment of sexual dysfunctions, is understanding people's approaches to the problem. This study was planned to investigate whether there is a relationship between self-esteem and body perception levels of patients with sexual dysfunction and preferential applications to urology or psychiatry outpatient clinics.

Methodology

The study included 125 patients who sought treatment at urology and psychiatry outpatient clinics and were diagnosed with erectile dysfunction (ED) or premature ejaculation (PE) as a result of clinical evaluation. Sociodemographic data forms, the International Erectile Function Index (IIEF-6), the Premature Ejaculation Diagnostic Tool (PEDT), the Body Image Questionnaire (BIQ), and the Self-Esteem Rating Scale (SERS), were administered to the patients.

Results

When the patients were evaluated according to their complaints, there was no difference between the groups in body image or self-esteem. However, when the preferential admissions were evaluated through outpatient clinics, the self-esteem and body perception levels were high in the patients who applied primarily to the psychiatry outpatient clinic (p = 0.032, p = 0.046).

Conclusion

Psychological factors may affect male sexual dysfunctions in treatment admissions. It is important that andrology and psychiatry doctors work in cooperation in the treatment of sexual dysfunctions.

## Introduction

The World Health Organization (WHO) asserts that sexual health encompasses more than just the absence of sexual dysfunction (SD). According to the WHO's definition, it encompasses physical, emotional, mental, and social well-being [1]. SD arises when sexual health is compromised at any level and can be either primary or secondary to other issues. SD can be congenital or acquired, as well as generalized or situational. The etiology of SD often involves a complex interplay of organic, psychogenic, and social factors.

Erectile dysfunction (ED) is characterized by the inability to achieve and/or maintain a penile erection sufficient for satisfactory sexual activity [2]. The reported prevalence, stratified by age, varies depending on the study design, the definition of erectile dysfunction, and the methodologies used. Typically, the prevalence ranges from 1% to 10% for men under 40 years old, 2% to 15% for men aged 40 to 49 years, 22% to 31% for men aged 50 to 69 years, 20% to 40% for those aged 60 to 69, and 50% to 100% for men older than 70 years [3]. Organic causes (such as vascular and neurogenic factors, endocrine system disorders, adverse drug effects, and structural abnormalities) and psychogenic causes may coexist in the etiology of ED. Psychological factors related to ED commonly include depression, performance anxiety, and limited sexual knowledge and experience [4].

Premature ejaculation, simply defined, refers to the inability to adequately control ejaculation and fully enjoy sexual interaction [2]. Premature ejaculation affects approximately 30% of men worldwide, but certain reviews have suggested that this figure could be as high as 75% [5].

Body perception and self-esteem levels are among the psychological factors that may be associated with SD. Body perception encompasses a multidimensional structure comprising perceptual, affective, and cognitive components, representing an individual's feelings about their body and thoughts about their behavior [6]. Self-esteem is defined as accepting and embracing one's abilities and powers as they are, alongside appreciating oneself through self-awareness and realistic evaluation. It involves feelings of love, respect, and trust toward oneself [7]. Previous studies conducted with individuals without sexual problems have shown that low body image [8,9] and low self-esteem have a negative impact on sexual activities and satisfaction [9-11]. Frederick and Essayli compiled and suggested that sexual orientation is a significant determinant of body image concerns among all men, based on five large-scale studies conducted on heterosexual and gay men in the US. They emphasized that body mass index is a strong determinant of body dissatisfaction in men and highlighted the importance of focusing on body image concerns among men [8]. In a study conducted by Lin and Lin on university students in Southern Taiwan, positive and significant relationships were found between body image perception and self-esteem, body image perception and sexual satisfaction, and self-esteem and sexual satisfaction. They also concluded that gender, body image perception, and self-esteem were the main predictors of sexual satisfaction among university students in Southern Taiwan [9]. In a study conducted by Wischmann et al. on infertile couples, decreasing tendencies were observed in both partners in the domains of self-esteem and relationship satisfaction [10]. Peixoto et al. analyzed the effect of sexual self-esteem on the relationship between sexual functionality and sexual satisfaction among heterosexual university students in Portugal and reported that sexual self-esteem partially mediated the relationship between sexual functionality and sexual satisfaction [11]. Furthermore, it has been reported that preventing ED contributes to increased self-esteem [12].

In summary, the etiology of SD involves a combination of organic and psychological factors. Discussing SD-related issues in Muslim countries such as Turkey can be a complex matter. While primary healthcare services are available in our country, individuals also have the option to seek direct examination by a specialist if they wish. This situation allows individuals to present directly to a psychiatrist or urologist. Therefore, this particular study provides an opportunity to explore how individuals perceive their SD in contrast to many other countries. Learning how individuals interpret SD complaints is important in terms of accurate information and appropriate management of the treatment process. Our study aims to explain why some patients primarily prefer urology outpatient clinics, while others prefer psychiatry outpatient clinics for treatment. The primary aim of the study was to investigate whether there is a difference in the relationship between erectile dysfunction and premature ejaculation and self-esteem and body image. The secondary aim was to examine whether the presence of a decrease in self-esteem and body image affects the choice of seeking medical attention at urology or psychiatry outpatient clinics.

## Materials and methods

Study population

The study included patients who presented to urology and psychiatry outpatient clinics between July 2019 and January 2020 with solely ED or PE. The patients were diagnosed with ED or PE by a urologist or psychiatrist after clinical evaluation. Patients requiring additional examination or treatment were referred to another specialist. The first admission clinic was considered for statistical analysis. Exclusion criteria included known psychiatric disorders, congenital morphologic anomalies, genital system deformities, a history of surgery, and the use of drugs that may affect sexual function, such as phosphodiesterase-5 inhibitors. Inclusion criteria required patients to be literate and heterosexual. All patients provided informed consent and completed the questionnaires themselves. The study was conducted in accordance with ethical standards, the 1964 Helsinki Declaration, and its later amendments. This study was approved by the Ufuk University Faculty of Medicine Ethics Committee (Date: July 3, 2019, No.: 20190703/4). Informed consent was obtained from all participants for the study.

A total of 149 patients were enrolled in the study. Eighteen patients with comorbid ED and PE and six patients who provided random responses were excluded from the analysis. Therefore, the analyses were performed on a total of 125 patients. Sociodemographic data, the International Index of Erectile Function (IIEF-6), the Premature Ejaculation Diagnostic Tool (PEDT), the Body Image Questionnaire (BIQ), and the Self-Esteem Rating Scale (SERS) were administered to the participating patients.

Scales

Sociodemographic Data Form

This form was created by the study team to collect information about the patients' age, years of education, preferred outpatient clinic presentations, and symptoms.

International Index of Erectile Function

The IIEF is a widely used questionnaire for assessing erectile function. It consists of 15 questions and is a reliable and multidimensional self-administered scale for clinical evaluation, diagnosis, and treatment of ED [13]. According to this scoring system, a score between 26 and 30 points indicates the absence of ED, while a score below 26 points indicates the presence of ED. Mild ED is defined by a score between 22 and 25 points; mild-moderate ED is between 17 and 21 points; moderate ED is between 11 and 16 points; and severe ED is a score of 10 points or below. The Turkish version of the IIEF was used in this study, which included questions 1, 2, 3, 4, 5, and 15 related to erectile function [14].

Premature Ejaculation Diagnostic Tool

This tool was developed to investigate the essential elements of premature ejaculation according to the DSM-IV-TR definition, such as the perception of inability to control ejaculation, discomfort, dissatisfaction with sexual intercourse, and problems between sexual partners. The PEDT consists of five questions related to ejaculatory control and sexual satisfaction, and respondents are asked to rate their experiences on a scale from 0 to 4 for each question. The total score ranges from 0 to 20, and higher scores indicate a higher likelihood of PE [15]. The Turkish version of the PEDT, which has undergone validity and reliability studies, was used in this study [16].

Body Image Questionnaire

The BIQ was used to evaluate body perception in the study. It was developed by Secord and Jourard [17] and adapted to Turkish society through a validity and reliability study [18]. The BIQ contains 40 items and aims to measure individuals' satisfaction with various body parts and functions, including sexual activity. Higher scores indicate a more positive evaluation.

Self-Esteem Rating Scale

Coppersmith [19] developed the SERS, which measures self-esteem. The scale has been adapted to the Turkish population [20] and measures self-esteem levels in children and adults. Higher scores indicate higher levels of self-esteem.

Statistical analysis

Statistical analysis was performed using the SPSS Statistics version 15.0 package program (IBM Corp., Armonk, NY). The significance level was set at P<0.05. Sociodemographic characteristics and nominal psychiatric diseases are presented as percentages. Numerical variables are presented as means and standard deviations, and categorical variables are presented as numbers and percentages. Normality analysis was conducted by examining skewness and kurtosis coefficients, which should fall within ±2. Comparisons between groups were analyzed with the independent two-sample t-test. Pearson's correlation was used to examine the relationship between numerical variables, as the parametric assumptions were met.

## Results

In our study, the data of 125 male participants were examined. Sixty-six participants primarily presented to the psychiatry outpatient clinic and 59 to the urology outpatient clinic. Sixty-four participants were evaluated as having PE and 61 as having ED. Thirty-three patients with PE participated in the study from the urology outpatient clinic and 31 from the psychiatry outpatient clinic. Twenty-six patients with ED were included in the study from the urology outpatient clinic and 35 from the psychiatry outpatient clinic. The average age, years of education, and scale scores of the participants are summarized in Table 1.

**Table 1:** Sociodemographic data of patients Note: (a) mean ± SD; (b) n, %.

Age^a^	29.88 (±5.29)
Years of education^a^	9.9 (±2.49)
First application	
Urology^b^	59 (47.2%)
Psychiatry^b^	66 (52.8%)
Sexual disorder	
Erectile dysfunction^b^	61 (48.8%)
Premature ejaculation^b^	64 (51.2%)
IIEF-6 score^a^	13.36 (±3.79)
PEDT score^a^	12.02 (±4.28)
BIQ score^a^	113.14 (±13.93)
SERS score^a^	67.15 (±9.89)

When evaluated in terms of psychiatry and urology applications, there was a difference between the groups regarding years of education. This difference was primarily due to the higher years of education of the participants who presented to psychiatry (p<0.001). There was no difference in the mean scores of the IIEF-6 and PEDT.

When evaluated in terms of BIQ and SERS, there was a significant difference between the groups (p=0.046 and p=0.032), and mean scores were higher in people who primarily presented for psychiatry (Table 2).

**Table 2:** Evaluation of data according to priority polyclinic applications Note: a mean ±SD. *p<0.05; **p<0.001.

	Urology (n: 59)	Psychiatry (n: 66)	p-value
Age^a^	29.49 (±5.32)	30.22 (±5.27)	0.44
Years of education^a^	8.88 (±2.63)	10.80 (±1.95)	<0.001**
IIEF-6 score^a^	13.88 (±3.55)	12.89 (±3.96)	0.147
PEDT score^a^	11.83 (±4.28)	12.18 (±4.31)	0.649
BIQ score^a^	100.50 (±12.96)	115.48 (±14.43)	0.046*
SERS score^a^	65.15 (±9.51)	68.93 (±9.95)	0.032*

When the data were examined according to the patients' symptoms, there was a significant difference in IIEF, PEDT scores, and years of education (p<0.001, p<0.001, p=0.035, respectively). However, there was no statistically significant difference in age, BIQ, and SERS scores (Table 3).

**Table 3:** Evaluation of data according to the type of sexual dysfunction Note. a mean ±SD. *p<0.05. **p<0.001.

	ED (n:61)	PE (n:64)	p-value
Age^a^	29.4 (±5.23)	30.32 (±5.34)	0.334
Years of education^a^	9.40 (±3.01)	10.35 (±1.74)	0.035*
IIEF-6 score^a^	10.27 (±2.40)	16.29 (±2.20)	<0.001**
PEDT score^a^	8.32 (1.90)	15.53 (2.63)	<0.001**
BIQ score^a^	113.93 (±14.73)	112.37 (±13.18)	0.534
SERS score^a^	67.67 (±9.99)	66.65 (±9.85)	0.568

Pearson's correlation was used to determine the correlation between the scales. According to the results, a moderate correlation (adjusted r=0.503, p<0.001) was found between BIQ and SERS. A weak correlation (adjusted r=0.308, p=0.002) was found between IIEF scores and SERS scores in patients presenting with ED symptoms. In patients presenting with PE symptoms, a moderate negative correlation (r= −0.410, p=0.001) between PEDT and BIQ scores and a weak negative correlation (r= −0.279, p=0.026) between PEDT and SERS scores were observed (Table 4).

**Table 4:** Correlations of the scales applied to the participants Note: *p<0.05; **p<0.01.

	BIQ score		SERS score	
	Adjusted r	p-value	Adjusted r	p-value
All patients				
IIEF-6 score	0.115	0.202	0.093	0.303
PEDT score	−0.194	0.03*	−0.147	0.103
Patients presenting with the complaint of ED				
IIEF-6 score	0.227	0.79	0.380	0.002**
PEDT score	−0.112	0.39	−0.075	0.566
Patients presenting with the complaint of PE				
IIEF-6 score	−0.308	0.013*	0.054	0.673
PEDT score	−0.410	0.001**	-0.279	0.026*

## Discussion

A primary healthcare system is also available in the Turkish health system. However, unlike many countries, people have the right to be examined directly by specialists if they wish. Therefore, people can present directly to a urologist or psychiatrist. Although this situation causes some difficulties in health services, it may be possible to understand how people interpret their SD. We believe that one of the important points in the treatment of SD is understanding people's approaches to the problem. Additionally, our study is, to the best of our knowledge, the first in the literature to examine SD through both body perception and self-esteem.

Sexuality is a complex aspect of human physiology and significantly impacts the quality of life. The nervous, cardiovascular, endocrine, and reproductive systems all play interrelated roles in healthy sexuality [21-23]. Problems in any of these systems, as well as psychosocial factors, can negatively affect an individual's sexual life. Therefore, male SD is not a one-dimensional problem, and psychological factors also contribute to the development and maintenance of SD. Until now, little attention has been given to how individuals perceive the problem in the treatment of SD. Hence, our study aimed to evaluate patients presenting to different clinics. When evaluating the results in terms of primary presentation to psychiatry or urology, no significant difference in age was found between the groups. However, a significant difference was observed in years of education, with patients presenting to psychiatry having a higher level of education. Sexuality is still a difficult topic to discuss openly in Turkey, and the higher preference for psychiatry among more educated individuals may be related to their ability to express themselves more easily.

Self-esteem and body perception are closely related concepts that influence each other in a cause-effect relationship. Studies have shown that negative body image is associated with low self-esteem [24,25], and both negative body image and low self-esteem are risk factors for psychopathology, including depression, anxiety, and eating disorders [26]. Recent meta-analyses have also emphasized the relationship between self-esteem and sexual health [27], and improvements in body image have been shown to positively impact sexual function [28]. In our study, a significant relationship was found between body perception and self-esteem, consistent with the literature. Individuals with negative beliefs about their bodies are expected to have low self-esteem. Conversely, individuals with low self-esteem may perceive their bodies more negatively than they actually are. In both cases, it can be expected that sexual health will be negatively affected.

Our study found no difference in symptoms or priority of presentation between psychiatry and urology outpatient clinics. This suggests that we obtained two homogeneous groups in terms of clinical symptomatology related to SD. Additionally, no difference was found in self-esteem and body image scores between men with PE and ED. Thus, patients may be more concerned about the presence or absence of a sexual problem rather than the specific type of problem.

However, higher scores on the BIQ and SERS were observed in patients presenting to psychiatry. In contrast, lower body perception scores were found in patients primarily presenting to urology. Body perception refers to the physical aspect of the problem as perceived by individuals, and it can be inferred that those who perceive the problem as primarily physical are present in urology. On the other hand, low self-esteem may lead to a reduced inclination to seek psychological help. Previous studies have shown that self-stigma associated with seeking psychological help is related to low self-esteem and lower rates of help-seeking [29]. In our study, the fact that patients with low self-esteem did not primarily consult psychiatry can be considered a consistent finding in this sense.

According to a review focusing on studies after 2010, psychological treatment for SD in women was emphasized, while combination therapies were recommended for men [30]. Even when significant biological problems are identified, psychological factors cannot be overlooked in SD. Psychological factors such as performance anxiety, family problems, misinformation about sexuality, and additional stressors play a role in the onset and maintenance of SD [30]. Therefore, studies that address both psychological and organic factors are crucial.

In our study, we aimed to evaluate the IIEF, PEDT, body image, and self-esteem of individuals with SD based on their preferential outpatient clinic presentations. In terms of our first hypothesis, no significant relationship was found between PE and ED and body image or self-esteem. Thus, our first hypothesis was not confirmed. However, self-esteem and body perception were higher in individuals who primarily presented to psychiatry, supporting our second hypothesis. These results suggest that individuals who perceive their problems as more physical tend to present to urology, while those with low self-esteem may be more likely to seek treatment from psychiatry.

Our study has several limitations. First, the absence of sexual dysfunction does not necessarily guarantee sexual satisfaction, and including a healthy control group for comparison would have been beneficial. Second, our study only focused on how individuals with sexual dysfunction interpreted the problem and did not assess the etiology of sexual dysfunction. It would have been valuable to examine the impact of additional factors such as BMI, comorbidities, and drug use on the results. Additionally, we did not evaluate the treatment processes of individuals after their application. Future studies with larger sample sizes should address these limitations to gain further insights into the topic.

## Conclusions

Our study focused on how individuals with SD interpret their problem rather than the underlying causes of SD. We examined whether individuals perceive the issue solely as a physical problem or if it is associated with their self-esteem. The choice of specialist during the initial presentation was considered predictable based on how patients approached the problem. As the main conclusion of our article states, we found that patients with both low body image scores and low self-esteem primarily refer to urology. We believe that this can contribute to a more accurate evaluation and treatment of SD. Furthermore, considering this information, there is a need for additional studies based on biopsychosocial models.

## References

[1] (2022). Sexual health and its linkages to reproductive health: an operational approach. https://apps.who.int/iris/handle/10665/258738.

[2] McCabe MP, Sharlip ID, Atalla E (2016). Definitions of sexual dysfunctions in women and men: a consensus statement from the Fourth International Consultation on Sexual Medicine 2015. J Sex Med.

[3] McCabe MP, Sharlip ID, Lewis R (2016). Incidence and prevalence of sexual dysfunction in women and men: a consensus statement from the Fourth International Consultation on Sexual Medicine 2015. J Sex Med.

[4] Figueroa G (2000). Revista chilena de neuro-psiquiatría. Kaplan and Sadock's Comprehensive Textbook of Psychiatry.

[5] El-Hamd MA, Saleh R, Majzoub A (2019). Premature ejaculation: an update on definition and pathophysiology. Asian J Androl.

[6] Cash TF (2004). Body image: past, present, and future. Body Image.

[7] Mann M, Hosman CM, Schaalma HP, de Vries NK (2004). Self-esteem in a broad-spectrum approach for mental health promotion. Health Educ Res.

[8] Frederick DA, Essayli JH (2016). Male body image: the roles of sexual orientation and body mass index across five national U.S. Studies. Psychol Men Masc.

[9] Lin H, Lin Y (2018). The study of body image, self-esteem and sexual satisfaction of college students in southern Taiwan. Univers J Educ Res.

[10] Wischmann T, Schilling K, Toth B, Rösner S, Strowitzki T, Wohlfarth K, Kentenich H (2014). Sexuality, self-esteem and partnership quality in infertile women and men. Geburtshilfe Frauenheilkd.

[11] Peixoto MM, Amarelo-Pires I, Pimentel Biscaia MS, Machado PP (2018). Sexual self-esteem, sexual functioning and sexual satisfaction in Portuguese heterosexual university students. Psychol Sex.

[12] Özkent MS, Hamarat MB, Taşkapu HH, Kılınç MT, Göger YE, Sönmez MG (2021). Is erectile dysfunction related to self-esteem and depression? A prospective case-control study. Andrologia.

[13] Rosen RC, Riley A, Wagner G, Osterloh IH, Kirkpatrick J, Mishra A (1997). The international index of erectile function (IIEF): a multidimensional scale for assessment of erectile dysfunction. Urology.

[14] Turunç T, Deveci S, Güvel S, Pekşircioğlu L (2007). Uluslararası cinsel işlev indeksinin 5 soruluk versiyonunun (IIEF-5) Türkçe geçerlilik çalışmasının değerlendirilmesi. Türk Üroloji Dergisi.

[15] Symonds T, Perelman MA, Althof S (2007). Development and validation of a premature ejaculation diagnostic tool. Eur Urol.

[16] Serefoglu EC, Cimen HI, Ozdemir AT, Symonds T, Berktas M, Balbay MD (2009). Turkish validation of the premature ejaculation diagnostic tool and its association with intravaginal ejaculatory latency time. Int J Impot Res.

[17] Secord PF, Jourard SM (1953). The appraisal of body-cathexis: body-cathexis and the self. J Consult Psychol.

[18] Hovardaoğlu S (1993). Vücut algısı ölçeği. Psikiyatri, Psikoloji, Psikofarmakoloji (3P) Dergisi.

[19] Coopersmith S (1967). The Antecedents of Self-Esteem.

[20] Onur E (1982). Self-esteem in children and its antecedents. Unpublished Master Thesis.

[21] Trajanovska AS, Vujovic V, Ignjatova L, Janicevic-Ivanovska D, Cibisev A (2013). Sexual dysfunction as a side effect of hyperprolactinemia in methadone maintenance therapy. Med Arch.

[22] Gerra G, Manfredini M, Somaini L, Maremmani I, Leonardi C, Donnini C (2016). Sexual dysfunction in men receiving methadone maintenance treatment: clinical history and psychobiological correlates. Eur Addict Res.

[23] Ujah GA, Nna VU, Agah MI, Omue LO, Leku CB, Osim EE (2018). Effect of quercetin on cadmium chloride-induced impairments in sexual behaviour and steroidogenesis in male Wistar rats. Andrologia.

[24] Green MA, Scott NA, Cross SE (2009). Eating disorder behaviors and depression: a minimal relationship beyond social comparison, self-esteem, and body dissatisfaction. J Clin Psychol.

[25] Green SP, Pritchard ME (2003). Predictors of body image dissatisfaction in adult men and women. Soc Behav Pers.

[26] Forman M, Davis WN (2005). Characteristics of middle-aged women in inpatient treatment for eating disorders. Eat Disord.

[27] Sakaluk JK, Kim J, Campbell E, Baxter A, Impett EA (2020). Self-esteem and sexual health: a multilevel meta-analytic review. Health Psychol Rev.

[28] Weaver AD, Byers ES (2006). The relationships among body image, body mass index, exercise, and sexual functioning in heterosexual women. Psychol Women Q.

[29] Lannin DG, Vogel DL, Brenner RE, Tucker JR (2015). Predicting self-esteem and intentions to seek counseling: the internalized stigma model. Couns Psychol.

[30] Brotto L, Atallah S, Johnson-Agbakwu C (2016). Psychological and interpersonal dimensions of sexual function and dysfunction. J Sex Med.

